# Response to peripheral immune stimulation within the brain: magnetic resonance imaging perspective of treatment success

**DOI:** 10.1186/s13075-015-0783-2

**Published:** 2015-10-19

**Authors:** Marina Sergeeva, Jürgen Rech, Georg Schett, Andreas Hess

**Affiliations:** Institut for Experimental Pharmacology, Friedrich Alexander University Erlangen-Nürnberg, Fahrstrasse 17, 91054 Erlangen, Germany; Department of Internal Medicine III, Friedrich Alexander University Erlangen-Nürnberg, Ulmenweg 18, 91054 Erlangen, Germany

## Abstract

Chronic peripheral inflammation in diseases such as rheumatoid arthritis leads to alterations in central pain processing and consequently to mood disorders resulting from sensitization within the central nervous system and enhanced vulnerability of the medial pain pathway. Proinflammatory cytokines such as tumor necrosis factor (TNF) alpha play an important role herein, and therapies targeting their signaling (i.e., anti-TNF therapies) have been proven to achieve good results. However, the phenomenon of rapid improvement in the patients’ subjective feeling after the start of TNFα neutralization remained confusing, because it was observed long before any detectable signs of inflammation decline. Functional magnetic resonance imaging (fMRI), enabling visualization of brain activity upon peripheral immune stimulation with anti-TNF, has helped to clarify this discrepancy. Moreover, fMRI appeared to work as a reliable tool for predicting prospective success of anti-TNF therapy, which is valuable considering the side effects of the drugs and the high therapy costs. This review, which is mainly guided by neuroimaging studies of the brain, summarizes the state-of-the-art knowledge about communication between the immune system and the brain and its impact on subjective well-being, addresses in more detail the outcome of the abovementioned anti-TNF fMRI studies (rapid response to TNFα blockade within the brain pain matrix and differences in brain activation patterns between prospective therapy responders and nonresponders), and discusses possible mechanisms for the latter phenomena and the predictive power of fMRI.

## Peripheral-to-central communication

In the last few decades, a large body of knowledge has been accumulated regarding bidirectional communication between the immune system and the brain (for review see [1–7], for historical overview of the discoveries see [8]). A new branch of research, psychoneuroimmunology has emerged primarily from the great interest in the role of the immune system in neuropsychiatric disorders, especially major depression. Much attention has been attracted following the discovery that increased peripheral inflammation is associated with depression and fatigue [3–6, 9–13]. Noteworthy is the frequent occurrence of depression in patients with rheumatoid arthritis (RA) and infectious and autoimmune diseases [14–16], and, inversely, there are elevated concentrations of inflammatory markers in medically healthy patients with major depression. These findings stimulated further investigation of mechanisms by which inflammatory information is transmitted from the periphery to the central nervous system (CNS). It is now known that proinflammatory cytokines, in particular interleukin (IL)-1, IL-6, and tumor necrosis factor (TNF) alpha, are important transmitters of this information. IL-6 and TNFα appeared to be the most consistently elevated cytokines in the studies conducted on depressive patients (see meta-analysis [10]) as well as in animal models of depression [17]. Released when confronted with an immune challenge—infection, injury, or stress—or externally administered (e.g., interferon alpha in the case of cytokine therapy), cytokines not only trigger cascades of defensive responses on molecular and cellular levels, but also act on an organismic level. The cytokines instruct the CNS how it should adapt its behavior to the altered conditions or, in other words, how to conserve energy for the purpose of optimal recovery [18]. Furthermore, cytokines mediate genetic factors [19] and social factors such as stress [5, 6, 11, 20–23] or “social pain” [24], also promoting the development of depression.

Peripheral cytokine signals reach the brain via three principal pathways—humoral, neural, and cellular—via several mechanisms [4, 5] including (adapted from [5]): passage through leaky regions in the blood–brain barrier at circumventricular organs; active uptake across the blood–brain barrier; the “neural route” via local actions at peripheral vagal nerve afferents that relay cytokine signals to relevant brain regions, including the nucleus of the solitary tract and hypothalamus, and directly at sensory neurons; activation of endothelial cells and perivascular macrophages in the cerebral vasculature to produce local inflammatory mediators such as cytokines, chemokines, prostaglandin E2 (PGE2), and nitric oxide (NO); and recruitment of activated monocytes/macrophages and T cells from the periphery. A potential gateway for immune cells into the brain has been discovered recently: functional lymphatic vessels lining the dural sinuses [25]. Within the brain, peripheral cytokines act on a central network of microglia, astrocytes, and neurons, which in turn produce cytokines [26]. This can amplify cytokine signals. Cytokines activate the hypothalamic–pituitary–adrenal axis, stimulating the production of corticotropin-releasing factor, adrenocorticotropic hormone, and cortisol [1], and influence many other physiological processes in the CNS. The cytokines alter the metabolism of neurotransmitters serotonin [27, 28], dopamine [28–31], glutamate [32, 33], which in turn leads to decreased production of the trophic or growth factors necessary for neurogenesis and neuroplasticity [6, 34, 35], or norepinephrine [5, 6]. Many studies demonstrated reduction of brain matter, especially in hippocampus [19, 35–37], but also different alterations in other areas of the brain [38–43] under continuously elevated IL, or changes in functional connectivity [43–48]. The latter can best be encompassed by functional neuroimaging methods and will be discussed in a separate section.

## Rapid onset of behavioral consequences

Multiple studies on humans and on experimental animals with elevated levels of proinflammatory cytokines in the periphery report in their subjects the so-called “sickness behavior” [49, 50]. This phenomenon has been well described and implicates, depending on the severity of the disturbance, such symptoms as fatigue, psychomotor slowing [22, 29], anxiety, anhedonia [51], cognitive dysfunction [52] (for review of the role of cytokines in maintaining the normal cognitive function see [53]), social withdrawal [54], sleep alterations, and loss of appetite, in different combinations. Notably, the sickness behavior develops very rapidly. Frenois et al. [55], using a range of behavioral tests, distinguished two phases and characterized their time development in mice injected with lipopolysaccharide (LPS). The authors have shown that sickness behavior peaked after 6 h, followed by the depressive-like behavior 24 h post LPS. The latter was paralleled by a decrease in cellular activities, particularly within the extended amygdala, hippocampus, and hypothalamus as shown by immunohistochemistry. Stone et al. [56] revealed that the exploratory movement activity of mice injected with LPS was reduced a mere 2 h post treatment. Similar timing was described in studies on healthy humans using, for example, *Salmonella typhi* vaccine as an inflammatory challenge [22, 29, 57–59]. Vaccination with *S. typhi* appears to be an appropriate model for investigation of depressive symptoms not superimposed by actual disease burden; it does not induce sickness. In these studies, deterioration of mood was observed starting within 3 h and lasting for at least 6 h after injection.

Normally the release of proinflammatory cytokines is adaptive and temporary, and so are its consequences as already described. Quite a different situation arises if a cytokine challenge becomes chronic; for example, in cases of chronic inflammatory diseases or permanent stress [18]. In these cases, in addition to the known primary symptoms, we can expect the development of clinically relevant psychiatric disorders such as major depression.

## Rheumatoid arthritis and TNFα

RA is a chronic autoimmune inflammatory disease severely affecting the joints. As the disease progresses, RA rapidly leads to destruction of cartilage and bone tissue, which is associated with pain, swelling, stiffness, and even immobility of the joints. Quality of life for people suffering from RA is dramatically diminished, and pain is the prevailing symptom of this disease.

Treatment of RA was extremely challenging before the development and introduction of drugs inhibiting biological activity of TNF (anti-TNF therapies). While affected joints express a multitude of inflammatory mediators, systemic inhibition of TNFα turned out to be a particularly successful therapeutic strategy [60–62]. This success is based in part on the efficient alleviation of joint inflammation upon disruption of the inflammatory cytokine network in the affected joint. However, it has always been surprising how quickly the patient condition improves taking into account that RA causes irreversible structural damage to the bones, the cartilage, and the joint innervation pattern [61, 63]. Nevertheless, amelioration of pain and improvement of personal subjective feeling set in soon after the start of TNF neutralization, long before inflammation decline becomes identifiable using common clinical tests.

This observation led to the idea that there must be an independent, faster impact of TNF inhibition on the CNS. Do anti-TNF therapies elicit pain-reducing effects in the central pain pathway? Consequently, functional brain imaging studies have been conceived to investigate changes in brain function—pain processing in particular—under TNFα inhibition.

## Functional neuroimaging: impact of cytokines on brain functions

Neuroimaging methods have brought new opportunities for linking fields of medicine operating at different levels; that is, immunology and neurology [19, 21, 24, 29, 48, 54, 57, 58, 64, 65] (for review see [38]). In particular, neuroimaging has revealed individual brain structures, but also complex neurocircuits in the CNS that appear to be modulated by cytokine signaling from the periphery; for instance, the anterior cingulate cortex (ACC), the basal ganglia, including nucleus accumbens, striatum, and substantia nigra, and the insular cortex. Subgenual ACC plays an important role in depression, and dorsal ACC in anxiety and alarm. Basal ganglia are essential for motor activity and also for motivation. The insular cortex is strongly linked to the emotional state (particularly its anterior, limbic-related portion), self-awareness, and empathy, and plays an important role in the regulation of the body’s homeostasis.

Vaccination with *S. typhi* was paralleled by increased activation in subgenual and dorsal ACC, as demonstrated by functional magnetic resonance imaging (fMRI), and a deterioration in mood and anxiety [57]. In subjects vaccinated with *S. typhi*, perturbed activity in the substantia nigra was associated with an increase in the amount of time required to solve a cognitive task, consistent with the notion that cytokines influence dopamine transmission [30, 51], and also correlated with increased levels of IL-6 in the blood [29]. Persons injected with *Escherichia coli* endotoxin exhibited increases in depressed mood and anhedonia over time, correlated with a significant reduction of activity in the ventral striatum, part of the reward circuit, evoked by monetary reward cues [51]. Women exposed to *E. coli* endotoxin became more vulnerable to social exclusion and, in a similarly structured fMRI study, exhibited increased activity in dorsal ACC and anterior insula paralleled to an elevation of IL-6 in blood and to a deterioration in mood [24]. Functional connectivity of the insula has been shown to increase under inflammatory conditions [47].

**Fig. 1:**
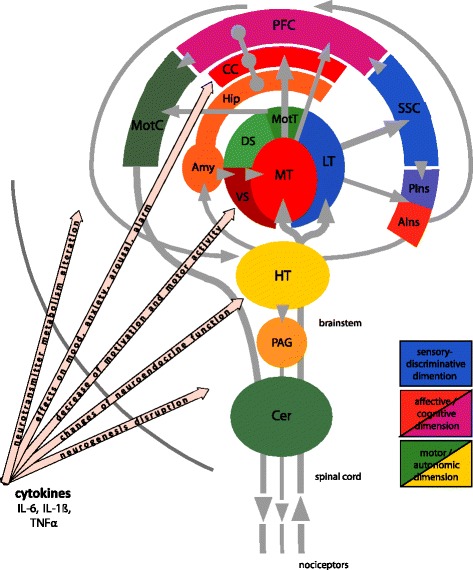
Schematic representation of the pain system and pathways for the influence of cytokines on brain function. Pain is a multidimensional sensation initiated at pain receptors in the periphery (nociceptors) by (potentially) harmful stimuli. The pain system consists of ascending and descending pathways which are highly interconnected at different processing stages up to the prefrontal cortex (*PFC*) as the highest station of nociceptive processing and a central hub of the cognitive dimension of pain. The most important transfer and “preprocessing” stations of nociceptive information are: the spinal cord (dorsal horn neurons), the brainstem including among others the medulla oblongata and peri-aqueductal grey (*PAG*), and the thalamus. From there and upwards one differentiates between two functionally overlapping but basically different subsystems. The lateral thalamus (*LT*) projects to the primary and secondary somatosensory cortices (*SSC*). These structures constitute the so-called lateral pain system responsible for the sensory-discriminative dimension of pain. The medial thalamus (*MT*) has tight connections to the anterior cingulate cortex (*CC*) and further to the PFC. These structures form the medial pain system considered to be in charge of the affective-motivational dimension of pain. Moreover, this system has extensive interconnections with the limbic system—entorhinal cortex, hippocampus (*Hip*), amygdala (*Amy*)—which is inseparably associated with emotions. The insular cortex has an intermediate position since it receives somatosensory input (posterior part, *PIns*), but has strong reciprocal connections to the amygdala (anterior part, *AIns*). Therefore, the insula can be ascribed to the medial pain system. As an “output” of pain processing, structures for immediate motor and autonomic responses and pain control are activated. Motor responses originate in the PFC, in higher order motor cortices and next in the primary motor cortex (*MotC*). They send downstream commands to the motor neurons in the spinal cord. The motor thalamus (*MotT*) and the motor basal ganglia dorsal striatum (*DS*) and cerebellum (*Cer*) take part in coordination of the motor responses. The other compartment of the basal ganglia, the ventral striatum (*VS*), belongs to an associative-limbic loop forming a link to the motor system influenced by motivational and emotional context. The hypothalamus (*HT*) orchestrates neuroendocrine and autonomic responses to pain. One of the most important elements of the descending inhibitory pain control acts via the PAG upon the dorsal horn neurons. Proinflammatory cytokines (IL-6, IL-1β, TNFα) reaching the brain exert powerful influence on the neurocircuits related to the affective-motivational dimension of pain and interfere with multiple physiologic processes relevant to mood regulation in the entire brain (see “Peripheral-to-central communication”). *IL* interleukin, *TNF* tumor necrosis factor

General evidence from functional neuroimaging suggests that two main mechanisms may be largely responsible for clinical pain in rheumatic diseases: CNS sensitization/impairment of inhibition; and alterations in the medial pain system (for review see [38]), which is responsible for the affective-motivational pain component (Fig. 1).

## fMRI prediction ahead of clinical evidence

Using fMRI, we [45] addressed the question of the rapid improvement of RA patients’ disease state following the start of TNF neutralization by anti-TNF. The rationale of the investigation was as follows. Given that TNFα also acts as a pain mediator, we hypothesized that during RA the cytokine constantly changes pain processing in the CNS. Systemic inhibition of TNFα should thus positively influence central pain processing, and this may happen long before it affects joint inflammation. Using blood oxygen level-dependent (BOLD) fMRI we measured the network of brain structures in RA patients activated in response to nociceptive stimulation of the affected joints before and at various points in time after intravenous infusion of infliximab, an anti-TNFα monoclonal antibody. Nociceptive activity in the brain pain matrix was significantly reduced as quickly as 24 h after TNF neutralization. This activity remained low until the end of the observation period 42 days after anti-TNF administration. BOLD activity decreases were observed in thalamus and in primary and secondary somatosensory cortices—structures responsible for the sensory-discriminative aspect of pain—but also to a large extent in parts of the limbic system, such as the cingulate and insular cortex, which are responsible for the affective-motivational or emotional pain component. Importantly, standard clinical measures of the disease activity—joint swelling and joint tenderness, composite disease activity scores (Disease Activity Score in 28 joints), and laboratory parameters such as the blood sedimentation rate and serum C-reactive protein and IL-6 levels—did not change within the first 24 h, but improved at a later stage of the treatment process. In contrast, subjective perception of pain in these patients, judged according to the visual analog scale (VAS), was ameliorated as early as 24 h after the first infusion, in parallel with the changes of nociceptive activity in the brain detected by fMRI.

These findings have been further substantiated by investigation into an animal model of arthritis: knockin mice overexpressing human TNFα (hTNFtg) [66]. Behavioral screening of these hTNFtg mice demonstrated that these animals develop characteristic signs of arthritis; for example, bone degeneration [61, 63] and decreased mobility [45]. Similar to humans, 24 h after treatment with infliximab arthritic mice showed significantly diminished sensitization to noxious stimuli, as has been established by von Frey as well as Hargreaves’ tests, which remained at the level of wild-type mice for at least 72 h. During the first 24 h following TNF neutralization, no apparent changes to clinic analog parameters or histopathologic signs of arthritis were observed. Remarkably, TNFα inhibition also completely restored motor activity (indicative of good mood), as demonstrated by the Rotarod test, within 24 h.

The fMRI part of the study in the hTNFtg mice was designed in a way which is possibly similar to the study of RA patients. As a response to nociceptive stimuli, before treatment, one could see significantly greater activation in the brain pain matrix of these mice compared with the wild-type mice. TNFα neutralization with infliximab led to a drastic reduction in this activity down to the level of wild-type mice (even lower in limbic areas) within 24 h. Again, there were profound decreases not only in the somatosensory cortex (sensory-discriminative aspect of pain), but also in expanded parts of the limbic system (emotional aspect).

Our research group [45] came to the conclusion that TNFα inhibition has a direct impact on central pain processing by far preceding its anti-inflammatory effects at the periphery. Suppression of neuronal activity in the limbic brain areas, in the cingulum and insula, could well explain the fast improvement of the subjective pain rating and overall feeling of RA patients following TNF neutralization. Therefore, one can speculate that anti-TNF also exhibits rapid antidepressant effects.

BOLD fMRI has been proven capable of issuing an early prediction of therapeutic success for TNF inhibition. This advantage of fMRI is especially valuable in light of the following dilemma: on the one hand, TNF blockers are expensive drugs with a potential risk for serious toxicity; and on the other, they have been shown to be highly effective, although not in all patients suffering from RA. To further investigate whether fMRI can also be used to differentiate between prospective responders and nonresponders to anti-TNF, we undertook a follow-up investigation [67]. In the follow-up investigation, RA patients received a standard injection of certolizumab-pegol, a subcutaneously administered anti-TNF, and fMRI measurements with the same stimulation paradigm as in the previous study [45] were performed before treatment and at several points in time after treatment. Clinical parameters were assessed at the same time points, as well as patients’ rating of global disease activity by VAS. In this study, similarly to clinical practice, one-half of the patients exhibited a significant clinically identifiable improvement after 28 days (responders), while the other half did not (nonresponders), although initial clinical baseline disease activity did not differ between the two groups. In accordance with the previous findings, the responder group demonstrated rapid (detectable on the third day) reduction of nociception-related activity in the brain pain matrix, which continued consistently over the following fMRI measurements. In the nonresponder group, in contrast, there was only a spurious decrease of the BOLD activity after 3 days, which then increased back to the initial levels after 7 and 28 days. A remarkable finding in this study was the striking difference in the number of significantly activated voxels under the nociceptive stimulation between the two groups before treatment: this number was high in the responders and low in the nonresponder group, particularly in the somatosensory, limbic, and associative brain areas. This study shed even more light on the predictive power of fMRI; the qualitative difference in the mean value of BOLD activation between the potential responders and nonresponders to anti-TNF was obvious even before treatment. As a next step, the quantitative determination of a threshold between responders and nonresponders is currently being performed in a clinical phase III multicenter study (PreCePRA NCT01864265).

## Discussion

Chronic inflammation occurring in RA and many other diseases triggers a continuous flow of afferent signals to the brain causing, among other things, chronic pain states. Central sensitization to these afferent stimuli and alterations in the medial pain system present as a result of chronic pain seem to be some of the main features of pathological sensory signaling in the CNS caused by inflammation [38]. RA is burdened with a profound affective-emotional aspect implicating fatigue, sleep impairment, and depressive mood. If TNF inhibitors exert, in addition to their anti-inflammatory effects at the periphery, a direct normalizing influence on central pain processing, then the fast relief reported by the patients following the commencement of anti-TNF therapy is not particularly surprising, since the standard clinical judgments in RA (VAS for pain, Disease Activity Score in 28 joints, Health Assessment Questionnaire and the quality-of-life instrument Short Form 36) are based on the patient’s subjective perception, rather than on objective parameters of the disease [68]. Objective measures of this direct normalizing influence were shown in the two clinical experimental studies [45, 67], albeit with a limited number of subjects included. The studies confirmed that neutralization of the proinflammatory cytokine TNFα rapidly improves the subjective state of the prospective responder RA patients and demonstrated that this improvement is associated with a drastic reduction in nociception-related activity within the brain pain matrix; the latter not only in the structures responsible for the sensory-discriminative component of pain, but importantly to a large extent also in the structures driving its affective-motivational aspect and involved in mood changes, including depression, and memory. BOLD fMRI of the brain has demonstrated potential ability to predict the therapeutic success of TNF inhibition at an early stage in the course of treatment [45] and possibly even before treatment is commenced [67]. One can hypothesize that the response to anti-TNF therapy depends on patients’ subjective disease perception which is reflected in the individual brain activity pattern. Could this statement equally be assigned to disorders other than RA caused by chronic inflammation?

Neutralization of TNFα was shown to be highly effective in treating the other complex diseases such as psoriasis [69] and Crohn’s disease [70–73], with a profound impact on depressive mood. Remarkably, in these diseases an improvement in the symptoms also occurs before inflammation remission becomes clinically identifiable. Psoriasis patients who received etanercept (a soluble TNF receptor that prevents TNFα-mediated responses by competitively inhibiting the interaction of TNF with cell-surface receptors) exhibited a significant improvement in fatigue and depression, the former correlated with and the latter not correlated with objective measures (skin clearance or joint pain) [69]. Etanercept also has been successful in reducing fatigue in cancer patients [74].

Fascinating parallels can be seen between the findings of the follow-up RA fMRI study [67] and a Crohn’s disease study by Atreya et al. [72]. The authors of the latter study managed an early prediction of the therapeutic success of adalimumab (TNF antibody) in patients with this disorder. Like in RA, in spite of the clinical efficacy of anti-TNF treatment, about 50 % of patients with Crohn’s disease do not respond to adalimumab, as determined by lack of a 100-point reduction of the clinical activity score (Crohn’s disease activity index) within 4 weeks after therapy initiation [70]. With the help of in-vivo imaging using a fluorescent TNF antibody, the researchers [72] visualized intestinal immune cells carrying membrane-bound TNF (mTNF). With respect to the number of mTNF(+) cells, the patients, as in the RA study, were split into two distinct clusters: in one of the clusters this number was large, and in the other the number of mTNF(+) cells was small. During the course of therapy, again as in the RA study, the former turned out to be responders—and their response was sustainable—and the latter turned out to be nonresponders. Moreover, in full accordance with the RA studies [45, 67], a recent fMRI investigation of Crohn’s disease patients [73] confirmed that TNF neutralization with infliximab rapidly improved the subjective state of the prospective responders and demonstrated that this improvement was paralleled by a drastic reduction in nociception-evoked activity within the brain pain matrix as early as 24 h after the first injection, again by far preceding peripheral anti-inflammatory effects such as mucosal healing.

If the patient’s reflections of the abovementioned disorders in the brain have an important contribution to the overall disease state, and TNF blockers do indeed exert quick antidepressant effects, then there is hope that fMRI could also help in predicting the anti-TNF treatment outcome in these diseases.

## Conclusion

CNS effects of cytokine blockers may, of course, differ among various diseases. Individual cytokine blockers might possess unique properties in modulating brain function; for example, IL-1 blockade leads to improvement of fatigue in patients with Sjögren’s syndrome and diabetes mellitus, whereas TNF blockade does not [75–77]. In contrast, despite effects on fatigue [78] and potent anti-inflammatory properties, IL-1 blockade has not resulted in comparable improvement of composite disease activity indices in RA as compared with anti-TNF treatment. It is plausible that each of these cases implicates unique changes in the brain pain matrix and would thus be differentially encompassed with fMRI. Furthermore, innate limitations of the BOLD fMRI method should also be taken into account. For instance, the technique cannot always strictly differentiate between the direct impact of a therapy on the affective-motivational aspect of pain processing and the “secondary” impact, mediated via the sensory-discriminative pain pathway. A degree of caution is required when interpreting the data. Nevertheless, it is more than tempting to explore the capabilities of the noninvasive, fast, and spatially highly resolved fMRI method to validate and to predict the therapeutic success over a broader range of chronic inflammatory disorders and cytokine-targeting therapies.

## Note

This article is part of the series *New technologies*. Other articles in this series can be found at http://arthritis-research.com/series/technology.

## Abbreviations

ACC: Anterior cingulate cortex
BOLD: Blood oxygen level dependent
CNS: Central nervous system
fMRI: Functional magnetic resonance imaging
hTNFtg: Knockin mice overexpressing human tumor necrosis factor alpha
IL: Interleukin
LPS: Lipopolysaccharide
mTNF: Membrane-bound tumor necrosis factor
NO: Nitric oxide
PGE2: Prostaglandin E2
RA: Rheumatoid arthritis
TNF: Tumor necrosis factor
VAS: Visual analog scale
